# Exercise-Induced Regulation of Bone Remodeling via Mitophagy: A Review of Current Evidence

**DOI:** 10.3390/biom16081171

**Published:** 2026-08-11

**Authors:** Sicheng Yan, Xinjia Li, Yu Yuan, Yongjie Yang, Xuewen Tian, Xi Chen, Lan Zhang, Shihua Zhang

**Affiliations:** 1 College of Sports and Health, Shandong Sport University, Jinan 250102, China; 2 School of Exercise and Health, Guangzhou Sport University, Guangzhou 510500, China; 3 School of Rehabilitation Medicine, Wenzhou Medical University, Wenzhou 325035, China; 4 Department of Sport and Recreation, Technological and Higher Education Institute of Hong Kong, Chai Wan, Hong Kong, China

**Keywords:** exercise, osteoporosis, mitophagy, bone remodeling, bone metabolism

## Abstract

Bone remodeling imbalance represents the fundamental pathological basis of osteoporosis. Exercise is broadly regarded as a valuable non-pharmacological strategy for preventing and managing osteoporosis; however, the precise molecular mechanisms through which exercise modulates bone metabolism remain incompletely understood. Mitophagy has recently been recognized as an important mediator linking exercise to the regulation of bone remodeling. This review centers on the “exercise–mitophagy–bone remodeling” axis, systematically outlining the biological processes and regulatory determinants of mitophagy within the bone microenvironment. Evidence suggests that mitophagy facilitates bone formation by preserving mitochondrial quality, attenuating oxidative stress, and optimizing cellular energy metabolism. Moreover, it exerts stage-specific inhibitory effects on bone resorption during osteoclast differentiation. Particular emphasis is placed on the mechanisms by which exercise activates mitophagy-related signaling pathways via metabolic, mechanical, and hypoxic stimuli. In addition, exercise may enhance the efficiency of this regulatory axis by maintaining vitamin D and calcium homeostasis and modulating estrogen signaling pathways. The differential effects of exercise modalities and durations on these processes are also critically evaluated. Finally, this review addresses current limitations in existing research and highlights future directions, including the optimization of exercise interventions targeting mitophagy and the integration of multi-omics approaches. These findings offer a theoretical basis for designing precise exercise regimens and combined therapeutic approaches in the management of osteoporosis.

## 1. Background

Osteoporosis (OP) is a highly prevalent chronic degenerative bone disease worldwide, characterized by decreased bone mineral density, increased bone fragility, and deterioration of bone microarchitecture. Its core pathogenic mechanism is the dynamic imbalance of bone remodeling [1]. Bone remodeling, a critical process for maintaining skeletal structural integrity and functional stability, depends on the dynamic equilibrium between osteoblast-driven bone formation and osteoclast-mediated bone resorption [2].

However, factors such as aging, estrogen deficiency (leading to a higher prevalence in postmenopausal women), and nutritional imbalance disrupt this equilibrium by triggering excessive accumulation of reactive oxygen species (ROS) and impaired cellular antioxidant capacity, which promote the production of inflammatory factors (TNF-α, IL-6) and mitochondrial dysfunction [3], leading to suppressed osteoblast function or abnormally enhanced osteoclast activity, thereby inducing osteoporosis [1]. The disease is often accompanied by pain and fragility fractures in multiple skeletal sites (e.g., spine, hip, and wrist), severely impairing patients’ quality of life. Furthermore, spinal deformities may result in visceral compression, motor dysfunction, and psychological disorders, forming a vicious cycle of “fracture–reduced mobility–further bone loss,” thus constituting a major hazard to the health of middle-aged and elderly individuals [4].

Exploring secure and efficient strategies to prevent and treat osteoporosis has become a core concern within the domain of bone metabolism. Currently, intervention strategies for osteoporosis mainly include nutritional supplementation, pharmacological therapy, and non-pharmacological approaches. Among these approaches, exercise is widely acknowledged as one of the most effective non-pharmacological measures, featuring benefits including low side effects, long-term sustainability, and positive impacts on general health. Exercise may modulate osteoblast proliferation and differentiation, and also affect osteoblast function, either directly or indirectly, by regulating key bone-associated signaling pathways, including the Wnt pathways. Exercise-induced Irisin activates autophagy by facilitating the formation of the Atg12–Atg5–Atg16L complex and promotes osteogenesis via the Wnt/β-catenin signaling pathway [5]. However, the detailed molecular basis underlying these effects is still not completely understood [6,7,8].

In recent years, mitophagy, a key process for maintaining mitochondrial homeostasis, has been identified as closely linked to the regulation of bone remodeling. Mitophagy exerts a critical regulatory function in the survival, differentiation, and functional homeostasis of osteoblasts and osteoclasts by eliminating impaired mitochondria, lowering ROS accumulation, and preserving steady energy metabolism [9,10]. Mechanical stress generated by exercise is capable of enhancing mitophagy in the bone microenvironment, which in turn induces adaptive mitochondrial alterations and modulates intercellular coupling. This forms a mechanistic axis centered on mitophagy, namely the “exercise–mitophagy–bone remodeling” pathway [11,12].

Relevant literature was searched using Google Scholar, PubMed, and CNKI databases. Literature retrieval was performed in December 2025 with the following keyword combinations: “physical activity/exercise/sport” + “vitamin D/estrogen/mechanical stress” + “mitophagy/mitochondria” + “osteoporosis/osteoblast/osteoclast/bone/bone remodeling”. A total of 254 potentially relevant articles were initially identified. Inclusion criteria: (1) Original research articles and authoritative reviews focusing on exercise, mitochondrial homeostasis, mitophagy and bone remodeling; (2) Studies conducted in humans or mammalian models; (3) Papers written in English or Chinese; (4) Classic foundational studies and recent high-quality publications. Exclusion criteria: (1) Meeting abstracts, case reports, reviews without original analysis, patents and dissertations without formal publication; (2) Studies based on non-mammalian species; (3) Literature irrelevant to the association between exercise, mitophagy and bone metabolism after full-text evaluation. After screening, retaining classic literature and integrating reviewer suggestions, 150 references were ultimately included in this review. It should be acknowledged that publication bias may exist, as studies with positive outcomes are more likely to be published, whereas negative or non-significant findings tend to remain unpublished.

## 2. Overview of Mitophagy

Autophagy is a highly conserved intracellular “self-renewal” mechanism that degrades misfolded proteins and damaged organelles through lysosomal pathways, thereby enabling material recycling and energy reutilization [13,14]. Mitochondria, as the central organelles responsible for energy metabolism—primarily through ATP production via oxidative phosphorylation—and the regulation of reactive oxygen species (ROS), play a decisive role in determining cell survival and physiological function [15,16].

Under normal physiological circumstances, mitochondrial quality is preserved via the dynamic equilibrium between fusion and fission. Nevertheless, when mitochondria suffer damage resulting from aging, external stress, or pathological triggers, they exhibit membrane potential depolarization, excessive ROS generation, and the release of pro-apoptotic proteins. These events can trigger oxidative stress cascades, ultimately leading to cellular dysfunction or even cell death [17].

As a major subtype of selective autophagy, mitophagy specifically denotes the process in which impaired mitochondria are selectively identified, sequestered, and broken down through the autophagic system. It serves as a central regulatory mechanism for sustaining mitochondrial homeostasis and cellular metabolic stability [18].

In the bone microenvironment, mitophagy plays a direct role in modulating bone remodeling by influencing the survival, differentiation, and functional activities of osteoblasts [19,20], osteoclasts [21,22], and bone marrow mesenchymal stem cells (BMSCs) [23,24,25,26]. As a result, mitophagy has become a key research area for exploring the pathogenesis of chronic degenerative diseases including osteoporosis, and identifying innovative therapeutic targets [9,10].

### 2.1. Biological Process of Mitophagy

**Figure 1:**
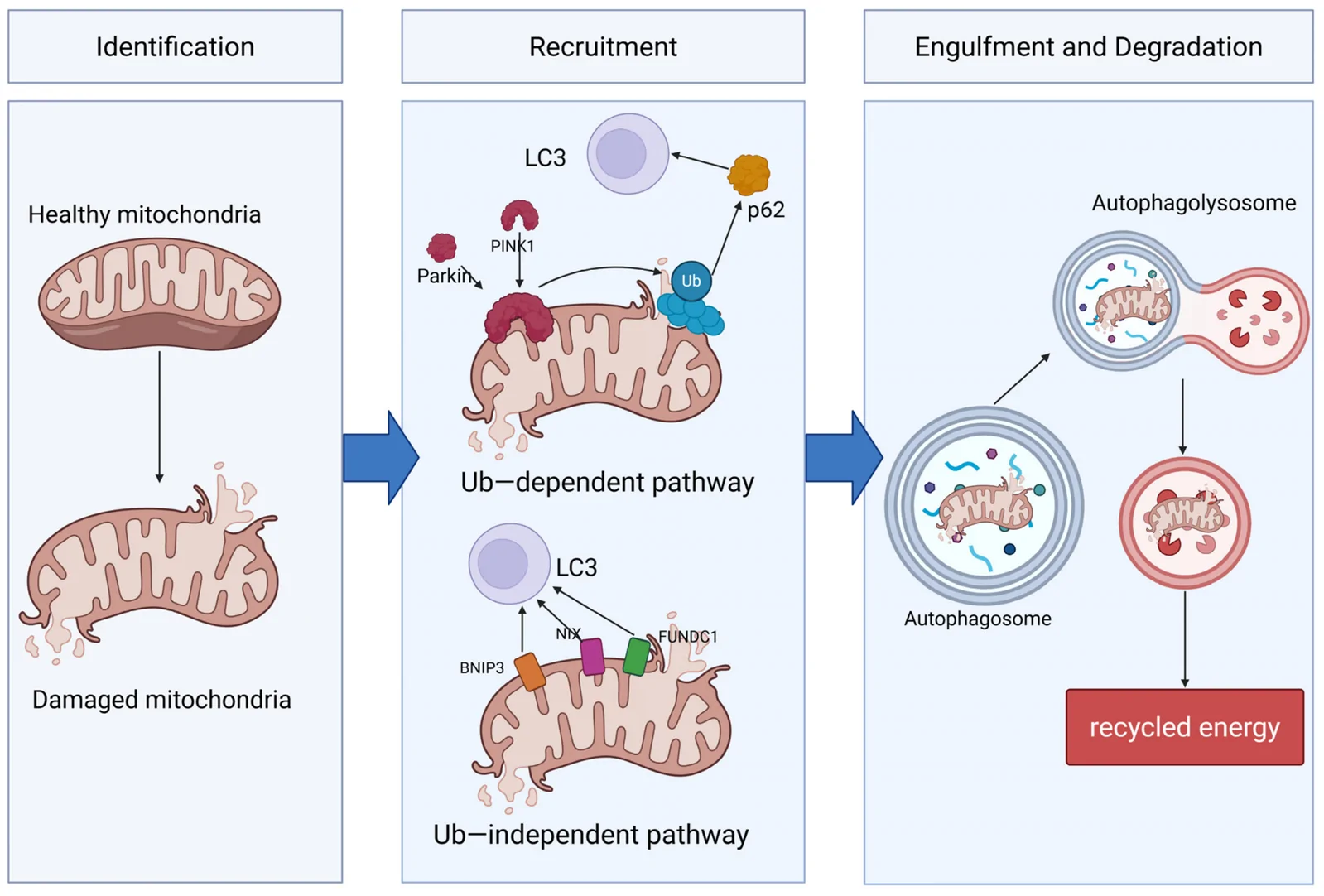
Biological Process of Mitophagy. This figure illustrates the biological process of mitophagy. The large connecting arrows represent the temporal progression across distinct mitophagy stages. Small arrows within the *Identification* and *Engulfment and Degradation* panels indicate temporal changes inside each respective stage, while small arrows in the *Recruitment* panel denote regulatory interactions between proteins. Healthy mitochondria become damaged and are subsequently targeted for degradation. In the ubiquitin (Ub)-dependent pathway, accumulation of PINK1 on the outer mitochondrial membrane leads to Parkin recruitment, thereby triggering ubiquitination of various mitochondrial proteins. Ubiquitin chains on damaged mitochondria recruit adaptor proteins such as p62, which interact with LC3 to initiate mitophagy. In the Ub-independent pathway, mitochondrial receptors including BNIP3, NIX, and FUNDC1 directly interact with LC3, thereby initiating the process of mitophagy. Damaged mitochondria are thereafter engulfed by autophagosomes, which subsequently fuse with lysosomes to be degraded. This process results in the recycling of mitochondrial components and the production of reusable energy.

Mitophagy consists of three essential steps: the identification of injured mitochondria, recruitment of autophagosomes, and degradation via lysosomes (Figure 1). Its molecular mechanisms are highly specific and involve complex regulatory networks.

During the initiation phase of mitochondrial damage and signaling, factors such as cellular stress, aging, and nutrient deficiency induce mitochondrial membrane potential depolarization [27,28]. The loss of membrane potential disrupts ionic homeostasis between the mitochondrial matrix and intermembrane space, while simultaneously promoting excessive ROS generation [27]. This further exacerbates mitochondrial damage and activates downstream autophagic signaling pathways.

During the selective recognition phase of damaged mitochondria, these organelles are specifically marked and identified by the dissipation of their mitochondrial membrane potential [28,29]. This process is mediated through both ubiquitin-dependent and ubiquitin-independent pathways. The ubiquitin-dependent pathway primarily involves the PTEN-induced kinase 1/Parkin (PINK1/Parkin) signaling axis [30], whereas ubiquitin-independent pathways include Bcl-2/adenovirus E1B 19 kDa-interacting protein 3/NIP3-like protein X (BNIP3/NIX) and FUN14 domain-containing protein 1 (FUNDC1) [31]. These upstream mitophagy pathways facilitate the recruitment of autophagosomes to engulf damaged mitochondria, forming mitophagy complexes that are subsequently transported to lysosomes [29].

Finally, in the stage of autophagosome engulfment and lysosomal degradation, mitophagosomes fuse with lysosomes, and their internal components are broken down by lysosomal hydrolases [28,29]. The resulting basic energy substrates are then recycled and reutilized by the cell [28].

### 2.2. Factors Influencing Mitophagy in the Bone Microenvironment

The bone microenvironment comprises osteoblasts, osteocytes, osteoclasts, BMSCs, immune cells, and the extracellular matrix, and its homeostasis depends on the coordinated regulation of multiple factors. Emerging evidence indicates that nutrients, hormones, mechanical stimuli, and pharmacological agents can regulate mitophagy within the bone microenvironment through specific molecular mechanisms, thereby influencing the balance of bone remodeling.

#### 2.2.1. Nutritional Factors

Vitamin D and calcium are essential nutrients for maintaining bone metabolic homeostasis. Vitamin D is converted into its active form, 1,25-dihydroxyvitamin D3 [1,25(OH)_2_D_3_], through two hydroxylation steps in the liver and kidneys. It then interacts with the vitamin D receptor (VDR), effectively promoting calcium reabsorption in both the intestinal mucosa and renal tubules [32,33,34], thereby promoting bone mineralization. Beyond their synergistic roles, vitamin D and calcium can independently regulate mitophagy. Vitamin D intervention has been shown to promote mitophagy in target cells [35,36,37,38,39,40]. Meanwhile, maintaining mitochondrial Ca^2+^ homeostasis has been demonstrated to significantly inhibit excessive mitophagy in osteoblasts in diabetic models, while upregulating osteogenic differentiation markers such as CTNNB1, Runx2, and BMP2, thereby alleviating osteoporosis [41].

**Figure 2:**
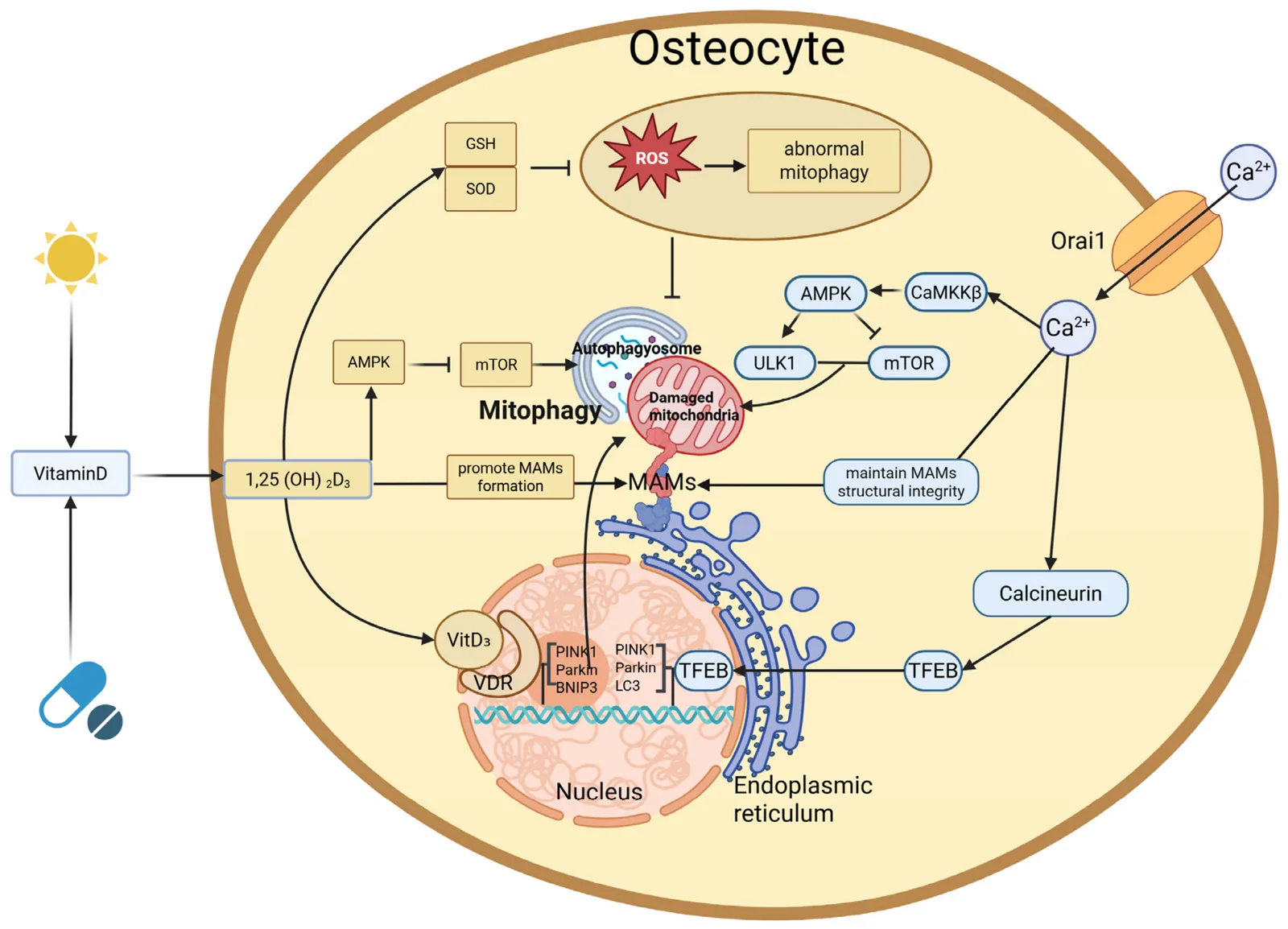
Vitamin D and Calcium Regulate Mitophagy. This figure shows how vitamin D and calcium regulate mitophagy in osteocytes. Arrows indicate the direction of signaling flow within regulatory pathways. Yellow-colored molecules represent components related to vitamin D signaling, while blue-colored molecules correspond to calcium-associated elements. The active form of vitamin D, 1,25(OH)_2_D_3_, binds to VDR to control the expression of mitophagy-related genes and reduce oxidative stress by limiting ROS. It also regulates mitophagy through the AMPK/mTOR/ULK1 pathway and promotes MAM formation. Meanwhile, calcium influx via Orai1 activates CaMKKβ, AMPK, and calcineurin, leading to TFEB activation and lysosomal function. Together, these pathways maintain mitochondrial quality and proper mitophagy.

Vitamin D regulates mitophagy through both direct and indirect mechanisms (Figure 2). (1) Direct regulation of mitophagy-related pathways: As a transcription factor, the vitamin D receptor (VDR) can directly bind to the promoter regions of PINK1, Parkin, and BNIP3, thereby upregulating their expression and promoting mitophagy mediated by the PINK1/Parkin [35,36,38] and BNIP3 pathways [37,42]. For example, in ovarian granulosa cells, 1,25 (OH)_2_D_3_ promotes mitophagy by upregulating the expression of PINK1 and BNIP3, thereby facilitating mitophagy [42]. Notably, the regulation of BNIP3 by vitamin D is bidirectional. Under hypoxic stress, vitamin D upregulates BNIP3 expression to enhance mitophagy and mitigate cellular damage. In contrast, under excessive stress conditions (e.g., ischemia–reperfusion injury), vitamin D suppresses BNIP3 expression to prevent excessive mitophagy-induced apoptosis [37,42]. This dual regulatory effect underscores the precise protective actions of vitamin D on cells and mitochondria, while whether this mechanism can maintain bone cell homeostasis requires further validation. (2) Indirect regulation of oxidative stress and mitochondrial function: Vitamin D exerts antioxidant effects by upregulating the expression of antioxidant molecules including superoxide dismutase (SOD) and glutathione (GSH), thereby lowering intracellular ROS levels in bone cells and avoiding the abnormal overactivation of mitophagy induced by excessive oxidative stress [43]. In addition, vitamin D can activate signaling pathways such as the AMPK–mTOR axis to balance mitophagy and mitochondrial biogenesis in hepatocytes, thereby maintaining mitochondrial quantity and functional stability [39]. Nevertheless, whether this pathway exerts identical effects in osteoblasts and osteoclasts remains to be further explored.

Homeostasis of cytosolic Ca^2+^ is crucial for osteogenic differentiation and the preservation of bone cell function, and its regulation of mitophagy is mainly mediated via Ca^2+^-dependent signaling pathways (Figure 2). (1) CaMKKβ–AMPK pathway: When extracellular Ca^2+^ influx (e.g., via Orai1 calcium channels) elevates cytosolic Ca^2+^ levels, Ca^2+^ activates CaMKKβ, which subsequently phosphorylates and activates AMPK. Activated AMPK promotes mitophagy by inhibiting the mTOR signaling pathway or directly phosphorylating ULK1 (autophagy-related kinase 1) [44,45]. For example, in osteoblasts, targeted Ca^2+^ supplementation enhances mitophagy via this pathway and reverses age-related impairments in osteogenic function [46]. (2) Calcineurin–TFEB pathway: Ca^2+^ can also activate calcineurin, leading to the dephosphorylation and nuclear translocation of transcription factor EB (TFEB), which upregulates the expression of mitophagy-related genes such as PINK1, Parkin, and LC3, thereby further enhancing mitophagy [47].

Furthermore, Ca^2+^ influences mitophagy by modulating the structural integrity of mitochondria-associated endoplasmic reticulum membranes (MAMs). As a critical “signaling hub” between the endoplasmic reticulum and mitochondria, MAMs regulate mitochondrial membrane potential through Ca^2+^ transfer. Vitamin D promotes the formation of MAMs, enhances Ca^2+^ transport from the endoplasmic reticulum to mitochondria, and subsequently activates mitophagy [48,49]. This underscores the synergistic role of vitamin D and calcium in regulating mitophagy within the bone microenvironment.

#### 2.2.2. Hormonal Factors

Estrogen is a key hormone that inhibits bone resorption and maintains bone mass, and its deficiency is the primary cause of postmenopausal osteoporosis [50]. Recent research has demonstrated that estrogen can regulate mitophagy within the bone microenvironment via multi-target mechanisms by binding to estrogen receptors (ERs), consisting of nuclear receptors ERα/ERβ and the membrane-localized receptor GPER [51,52,53].

The regulation of mitophagy by estrogen is characterized by a “multi-receptor, multi-pathway” pattern [54]. Its core mechanisms involve the activation of the PINK1/Parkin and SIRT1/AMPK pathways, leading to positive regulation of mitophagy in bone cells, with clear cell-type-specific effects.

First, estrogen primarily exerts transcriptional regulation through nuclear estrogen receptors α (ERα) and β (ERβ). As nuclear receptors, ERα/β are capable of directly binding to the promoter regions of PINK1, Parkin, and BNIP3 genes, which in turn upregulates their expression and facilitates mitophagy mediated by the PINK1/Parkin and BNIP3 pathways [55,56,57] For example, in osteoblasts, 17β-estradiol (the biologically active form of estrogen) enhances mitophagy by upregulating PINK1 expression via ERα, facilitating the elimination of damaged mitochondria, mitigating ROS accumulation, and ultimately contributing to the promotion of osteogenic differentiation [55]. In chondrocytes, 17β-estradiol activates the AMPK signaling pathway through ERs, enhancing mitophagy to alleviate inflammation-induced mitochondrial damage [58].

In addition to the regulatory effects mediated by nuclear receptors, estrogen can further trigger rapid non-genomic responses via GPER. Upon activation, GPER promotes ULK1 phosphorylation through ERK1/2 or AMPK signaling pathways, thereby enhancing mitophagy [53,59,60,61]. Meanwhile, GPER can inhibit the PI3K/Akt/mTOR pathway, inhibiting cell apoptosis induced by excessive mitophagy [62,63,64].

Furthermore, estrogen indirectly regulates bone cell apoptosis and differentiation. On the one hand, it promotes mitophagy and osteogenic differentiation of BMSCs via activation of the MAPK/Akt pathway. On the other hand, it inhibits macrophage polarization toward the osteoclast-like M1 phenotype, thereby reducing bone resorption [52]. In addition, estrogen suppresses the expression of RANKL in osteoclasts, indirectly reducing mitophagy activity and inhibiting osteoclast differentiation [65].

Moreover, estrogen-related receptors (ERRs), which can compete with ERs for estrogen response elements, have been shown to promote mitophagy by enhancing the transcriptional activity of TFEB [66], offering a novel potential target for the regulation of mitophagy mediated by estrogen.

#### 2.2.3. Pharmacological Factors

Pharmacological agents also exert a significant regulatory role in modulating mitophagy within the bone microenvironment. Recently identified compounds, such as the flavonoid Kaempferol [26], the novel pyrimidine derivative Pym-18a [20], and the sesquiterpene lactone Brevilin A [22], as well as traditional Chinese medicine formulations such as Buzhong Yiqitang [67] and Guilu Erxian [68], have all been shown to improve bone microenvironment function by promoting mitophagy.

#### 2.2.4. Mechanical Stimuli Factors

Besides nutritional and pharmacological factors, exercise has been extensively proven to exert adaptive and ubiquitous effects on mitochondrial biogenesis and functional activities [69]. As the most effective non-pharmacological strategy for preventing and treating osteoporosis, one of its core mechanisms involves activating mitophagy in the bone microenvironment through mechanical stress (e.g., fluid shear stress and tensile strain), metabolic stress (e.g., ATP consumption), and hypoxic stress (e.g., ROS generation). This ensures sustained mitochondrial integrity and stable bone cell function [70,71,72].

## 3. Mitophagy and Bone Remodeling in the Bone Microenvironment

Bone remodeling serves as a foundational process that preserves the structural integrity and functional stability of the skeleton in the bone microenvironment, and its homeostasis relies on the dynamic equilibrium between bone formation and bone resorption. Osteoblasts mediate bone formation by secreting osteocalcin, type I collagen, and alkaline phosphatase to facilitate bone matrix mineralization, whereas osteoclasts are responsible for bone resorption by secreting H^+^ to acidify bone minerals and releasing cathepsin K (CTSK) to degrade the bone matrix [73,74,75,76]. Various pathological factors, such as aging, estrogen deficiency, and nutritional imbalance, can disrupt this balance by suppressing bone formation and enhancing bone resorption, ultimately impairing bone remodeling homeostasis [1]. As a critical mechanism for sustaining mitochondrial homeostasis, mitophagy modulates the differentiation and functional activity of osteoblasts as well as osteoclasts. It exerts a bidirectional regulatory effect on bone remodeling by simultaneously promoting bone formation and inhibiting bone resorption. Notably, this regulatory role exhibits clear cell-specific and stage-specific characteristics.

### 3.1. Mitophagy Regulates Bone Formation

**Figure 3:**
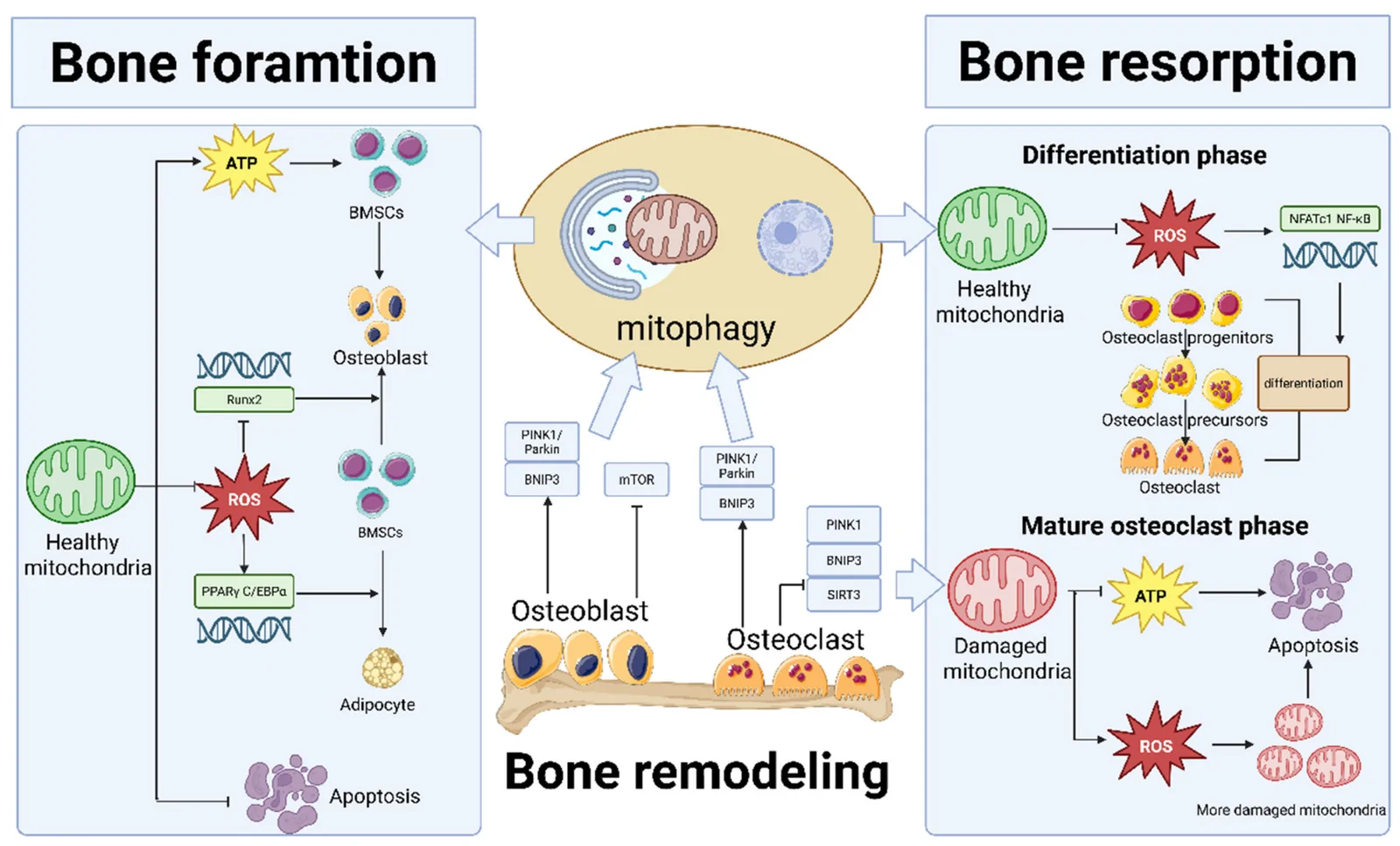
Regulatory mechanisms of mitophagy during bone remodeling. This figure shows the regulatory effect of mitophagy in bone remodeling. Arrows represent stimulatory/promoting effects, and blunt-ended symbols indicate inhibitory effects. The (**left**) panel shows bone formation: healthy mitochondria accelerate the differentiation and maturation of BMSCs into osteoblasts by supplying ATP, whereas ROS inhibit osteogenesis and promote adipogenesis and apoptosis. The (**right**) panel depicts bone resorption: under physiological conditions, healthy mitochondria suppress osteoclast differentiation by decreasing ROS production through mitochondrial mitophagy; when mitochondria are damaged, elevated ROS levels enhance mature osteoclast activity and apoptosis. The central panel illustrates that mitophagy modulates the functions of osteoblasts and osteoclasts via signaling pathways including PINK1/Parkin, BNIP3, and mTOR, thereby sustaining the dynamic balance between the formation and resorption of bone.

Mitophagy supports bone formation by preserving mitochondrial quality, regulating oxidative stress, and optimizing cellular energy supply (Figure 3). These effects are achieved through the coordinated and compensatory regulation of multiple signaling pathways.

#### 3.1.1. Mitophagy Maintains Mitochondrial Quality and Energy Homeostasis

Mitochondria act as the “energy center” for osteogenic differentiation of BMSCs and maturation of osteoblasts, and mitochondrial dysfunction can directly trigger apoptosis and suppress osteogenic differentiation. Studies have shown that apoptotic factors released from impaired mitochondria—including cytochrome c and Smac—as well as excessive ROS generation, are key factors contributing to BMSC apoptosis and the impairment of osteoblast mineralization ability [77,78]. Maintaining mitochondrial quality and energy homeostasis is crucial for sustaining the viability of bone cells and facilitating bone formation. On the one hand, mitophagy selectively sequesters and degrades depolarized and damaged mitochondria through signaling pathways including PINK1/Parkin and BNIP3/NIX, which in turn attenuates the liberation of apoptotic factors and decreases the apoptosis rates of BMSCs and osteoblasts [19,20,23,24,79,80,81,82,83,84,85]. On the other hand, mitophagy preserves the integrity of mitochondrial oxidative phosphorylation, ensuring continuous ATP production. In the process of BMSC osteogenic differentiation, the expression of osteogenic factors (e.g., Runx2 and Osx) as well as the synthesis of bone matrix demand a considerable amount of ATP. By optimizing mitochondrial quality, mitophagy provides a stable energy supply to support these processes [18,86].

#### 3.1.2. Mitophagy Guides the Osteogenic Differentiation of BMSCs Through Regulating ROS Levels

ROS levels serve a pivotal function in determining the differentiation fate of BMSCs [87,88]. Physiological levels of ROS can activate key osteogenic signaling pathways, whereas excessive ROS induces oxidative damage to DNA and proteins, downregulating the activity of osteogenic transcription factors like Runx2, and upregulating adipogenic markers including PPARγ and C/EBPα, thereby promoting adipogenic differentiation of BMSCs [89,90].

Under different physiological and pathological conditions, mitophagy achieves precise regulation of ROS levels through three major signaling pathways, thereby guiding BMSCs toward osteogenic differentiation.

The PINK1/Parkin pathway acts as the main initiator of mitophagy in osteoblasts, and its dysfunction directly causes mitochondrial damage and impaired bone formation [23,24,79,83,91]. Lee et al. [79] found that PINK1 deficiency gave rise to substantial bone loss in an ovariectomized (OVX) mouse model. Knockdown of PINK1 in osteoblasts caused a significant decline in the membrane potential of mitochondria, a 2.3-fold increase in ROS levels, and a 40–60% reduction in osteogenic markers, including alkaline phosphatase and osteocalcin. Mechanistically, PINK1 phosphorylates mitochondrial membrane proteins (e.g., TOM20) and ubiquitin, thereby recruiting Parkin to damaged mitochondria and initiating their autophagic degradation, ultimately reducing ROS production [92]. In addition, suppression of the PI3K/mTOR pathway can activate the PINK1/Parkin signaling axis, thereby attenuating oxidative stress-induced senescence in BMSCs, which further supports its protective function during osteogenesis [93].

The mTOR (mammalian target of rapamycin) signaling pathway regulates mitophagy in a bidirectional manner and serves as a critical node for environment-dependent regulation. Its effects vary depending on the cellular context. Under physiological or oxidative stress conditions, inhibition of mTOR relieves its suppressive phosphorylation of ULK1, thereby activating ULK1-mediated mitophagy and reducing ROS accumulation. For example, in H_2_O_2_-induced BMSC senescence models, mTOR inhibition decreases ROS levels and upregulates the osteogenic marker Runx2 [93]. On the contrary, in the context of pathological states, the activation of mTOR signaling has been demonstrated to enhance the expression levels of PINK1 and LC3-II in osteoblasts derived from osteoporotic mice, which in turn enhances mitophagic activity [94]. Further studies indicate that mTOR activation also facilitates the expression of PGC-1α and TFAM, which correlates with the biogenesis of mitochondria. This suggests a dual regulatory mechanism that involves both the clearance of damaged mitochondria and the synthesis of new mitochondria, thereby restoring mitochondrial function [95].

Under normal physiological conditions, the PINK1/Parkin pathway serves as the primary mechanism for eliminating damaged mitochondria and lowering ROS levels [79,81]. However, in aged BMSCs or under pathological conditions such as hyperglycemia and hypoxia, excessive ROS can suppress PINK1/Parkin activity [96]. In such cases, the ROS–HIF-1–BNIP3/NIX pathway acts as a compensatory mechanism to maintain mitophagy. Elevated ROS levels upregulate the expression of hypoxia-inducible factor-1α (HIF-1α), which functions as a transcription factor by binding to the promoter regions of BNIP3/NIX genes, thereby upregulating their expression. BNIP3/NIX then interacts with the autophagosomal membrane protein LC3 to mediate the degradation of damaged mitochondria. Although this pathway was initially reported to promote osteoclast differentiation [97,98], recent studies have demonstrated its protective role in osteoblasts. Qiao et al. [80] demonstrated that lentivirus-mediated overexpression of HIF-1 in osteoblasts upregulated BNIP3/NIX expression, enhanced mitophagy activity, reduced ROS levels, and significantly improved osteogenic differentiation capacity. Moreover, as opposed to the control group, the BMD of ovariectomized (OVX) mice was significantly elevated. These findings suggest that, in both primary osteoporosis and secondary osteoporosis (e.g., diabetes-associated osteoporosis), pharmacological targeting of the ROS–HIF-1–BNIP3/NIX pathway may represent a promising therapeutic strategy.

Collectively, mitophagy precisely modulates intracellular ROS levels via three major pathways—PINK1/Parkin, mTOR, and ROS–HIF-1–BNIP3/NIX—to direct BMSCs toward osteogenic differentiation, while the PINK1/Parkin pathway plays a dominant role, mTOR mediates bidirectional regulation, and BNIP3/NIX acts as a key compensatory mechanism under pathological conditions. This precise, multilayered network is essential for bone homeostasis and provides promising therapeutic targets for osteoporosis.

### 3.2. Mitophagy Inhibits Bone Resorption

The effects of mitophagy on bone resorption exhibit pronounced stage-specific characteristics during osteoclast differentiation. During the differentiation phase, activation of mitophagy suppresses osteoclastogenesis, whereas in mature osteoclasts, inhibition of mitophagy induces apoptosis, thereby enabling precise regulation of bone resorption (Figure 3).

Osteoclast differentiation depends on moderate ROS signaling. ROS generated from mitochondrial dysfunction can activate key osteoclastogenic pathways, including the Ca^2+^/calcineurin–NFATc1 axis and NF-κB signaling. Mitophagy suppresses this differentiation cascade by reducing ROS levels [99,100]. The mediation of this process is primarily accomplished via the PINK1/Parkin and BNIP3 pathways.

The PINK1/Parkin pathway represents the principal regulator of mitophagy throughout osteoclast differentiation, and its deficiency results in excessive osteoclastogenesis. Jang et al. [101] reported in PINK1 knockout mice that the proportion of depolarized mitochondria and ROS levels in bone marrow macrophages (BMMs) were significantly increased, accompanied by enhanced osteoclast formation and enlarged resorption pit areas compared with wild-type mice. Notably, supplementation with the mitochondria-targeted antioxidant MitoQ markedly reduced osteoclast formation and bone resorption activity. Mechanistically, PINK1 phosphorylates Parkin, thereby activating its E3 ubiquitin ligase activity; this process further induces the ubiquitination of mitochondrial surface proteins (e.g., VDAC1), promotes the recruitment of autophagosomes, and facilitates the degradation of damaged mitochondria. This reduces ROS release and subsequently inhibits NFATc1 nuclear translocation as well as the expression of CTSK and TRAP [101].

In addition, the BNIP3 pathway serves as a key regulatory target under hypoxic conditions. Hypoxia is a common stress state in the bone microenvironment (e.g., during fracture healing or at high altitude) and is also an important factor promoting osteoclast differentiation [99]. Under hypoxia, PINK1/Parkin pathway activity is attenuated, and BNIP3 becomes the dominant regulator of mitophagy and osteoclastogenesis. Hypoxia induces HIF-1 expression, which upregulates BNIP3 transcription. BNIP3 binds to Beclin-1 through its BH3 domain, thereby dissociating Beclin-1 from Bcl-2-mediated suppression and facilitating mitophagy [97,98,102,103]. Conversely, inadequate BNIP3 expression gives rise to the buildup of damaged mitochondria, excessive ROS generation, and further activation of signaling pathways involved in osteoclast differentiation [22,97].

In contrast to the differentiation stage, the bone-resorbing function of mature osteoclasts depends on sustained mitophagy. On the one hand, mitophagy maintains ROS homeostasis and energy supply by clearing damaged mitochondria; on the other hand, inhibition of mitophagic activity leads to the accumulation of damaged mitochondria, triggers cytochrome c release, initiates apoptosis, and ultimately suppresses bone resorption [21]. This regulatory mechanism is closely associated with mitochondrial homeostasis mediated by sirtuin 3 (SIRT3). SIRT3, a mitochondrial matrix deacetylase, promotes mitophagy in mature osteoclasts by activating the PINK1/Parkin pathway [104] and maintaining mitochondrial function [105]. Studies have shown that in SIRT3 knockout mice, mitophagy activity in mature osteoclasts is reduced by 50%, apoptosis rates increase by 35%, and the number of resorption pits decreases by 40%. Moreover, deficiency of SIRT3 significantly weakens the bone loss triggered by estrogen deficiency in ovariectomized (OVX) mouse models [104,105]. These findings suggest that SIRT3 acts as a key activator of mitophagy in mature osteoclasts. Targeting SIRT3 inhibition may therefore represent a potential therapeutic approach for osteoporosis linked to excessive osteoclast activation, including postmenopausal osteoporosis.

Taken together, during the osteoclast differentiation stage, mitophagy is actively activated through the PINK1/Parkin and BNIP3 pathways, which reduces ROS levels and thereby suppresses osteoclastogenesis. In contrast, in mature osteoclasts, downregulation of PINK1, BNIP3, and SIRT3 impairs mitophagic activity, ultimately inducing osteoclast apoptosis. This stage-specific and precise regulatory mode reveals the bidirectional regulatory role of mitophagy in bone homeostasis, holding great promise for providing novel strategies to suppress excessive bone resorption and prevent and treat postmenopausal osteoporosis as well as other related bone diseases.

## 4. Role of Mitophagy in Exercise-Regulated Bone Remodeling

Exercise, serving as a core non-pharmacological intervention, acts as a regulator in bone remodeling, exerts its effects primarily through the dynamic modulation of mitophagy homeostasis, thereby facilitating bone formation and/or suppressing bone resorption. This regulatory process is achieved through three major pathways. First, exercise-induced metabolic stress and mechanical stress activate mitophagy-related signaling pathways. Second, hypoxic stress induced by exercise triggers adaptive mitophagy within the bone microenvironment. Third, exercise enhances the regulatory efficiency of mitophagy on bone cell function by modulating vitamin D/calcium homeostasis and estrogen signaling.

### 4.1. Exercise Regulates Mitophagy to Influence Bone Remodeling

**Figure 4:**
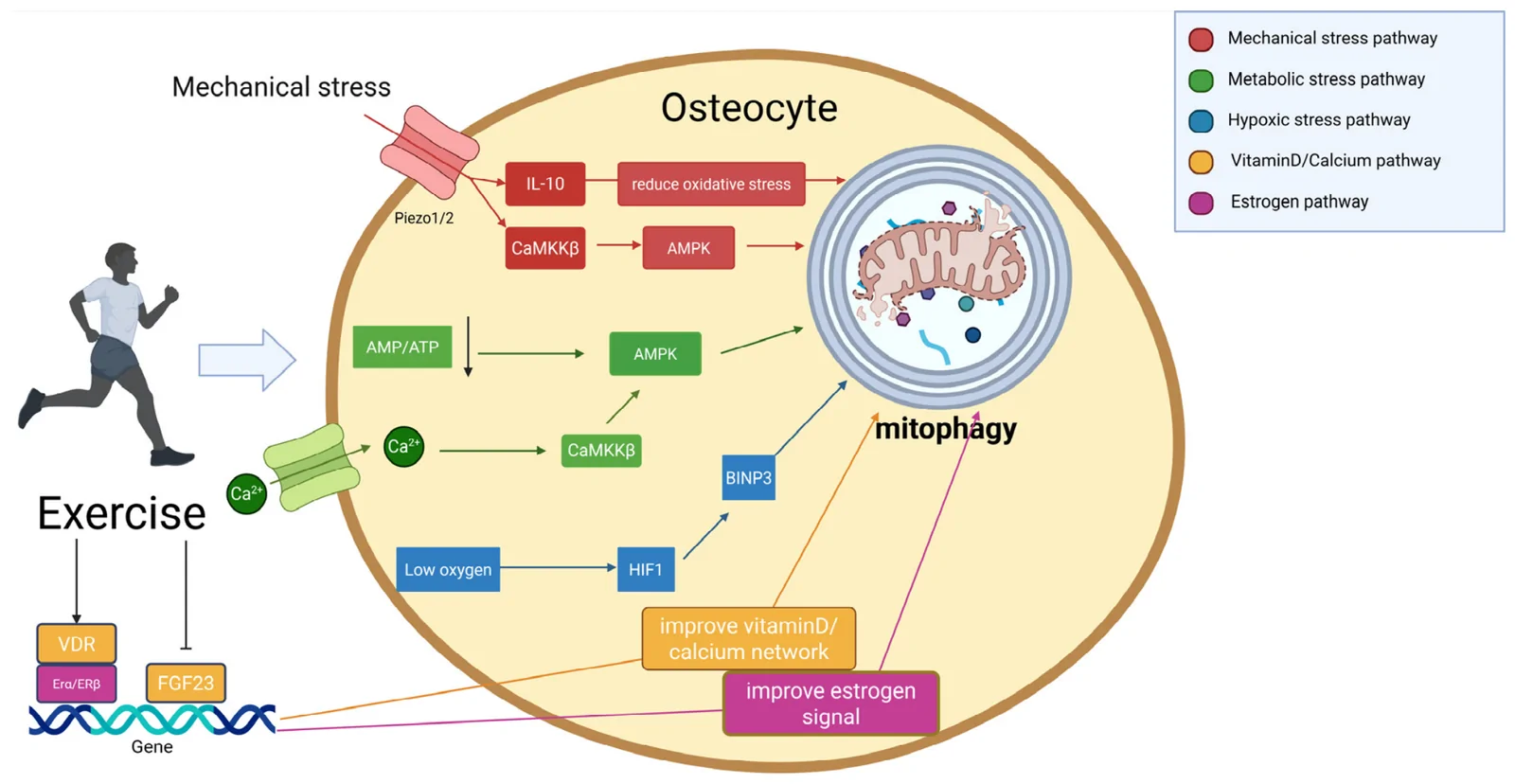
Exercise regulates mitophagy pathways. This figure illustrates how exercise promotes mitophagy in osteocytes through multiple pathways. Arrows represent stimulatory/promoting effects, and blunt-ended symbols indicate inhibitory effects. Different colors denote distinct signaling pathways, as illustrated in the embedded legend within the figure. Mechanical stress, metabolic changes (AMP/ATP, Ca^2+^), and hypoxia stress activate AMPK and HIF-1 signaling, thereby enhancing mitophagy. Exercise also improves the vitamin D/calcium network and estrogen signaling, further supporting mitochondrial quality control. Together, these pathways coordinate to maintain osteocyte function and bone health.

The regulatory effects of exercise on mitophagy depend on stress signal sensing, pathway activation, and mitochondrial quality remodeling (Figure 4). Fundamentally, exercise modulates mitophagy through three parallel signaling pathways. Moreover, these regulatory effects exhibit stage-specific differences between acute and long-term exercise. Through the coordinated regulation of mitophagy and mitochondrial biogenesis, physical activity ultimately enhances mitochondrial function in bone cells, thereby helping to maintain the homeostasis of bone remodeling.

#### 4.1.1. Metabolic and Mechanical Stress Modulate the Homeostasis Between Mitophagy and Mitochondrial Biogenesis via the AMPK Signaling Pathway

Metabolic stress represents a common mechanism by which various forms of exercise induce mitophagy. The key signaling hubs involved in this process are AMP-activated protein kinase (AMPK) and peroxisome proliferator-activated receptor gamma coactivator-1α (PGC-1α), which work together to regulate the balance between mitophagy and mitochondrial biogenesis [106]. During exercise, energy consumption in bone cells and BMSCs increases markedly, leading to an elevated intracellular AMP/ATP ratio that directly activates AMPK [45,107]. Meanwhile, exercise-induced calcium influx activates calcium/calmodulin-dependent protein kinase kinase β (CaMKKβ), and this activation further promotes AMPK activity through the Ca^2+^/CaMKKβ/AMPK signaling pathway [45,108].

Activated AMPK regulates mitophagy through two primary mechanisms. On the one hand, it directly phosphorylates the key autophagy-initiating protein ULK1, thereby relieving the inhibitory effect of mTOR on ULK1 and initiating the mitophagic process [71,109]. On the other hand, AMPK phosphorylates PGC-1α, enhancing its transcriptional activity. PGC-1α not only upregulates mitochondrial transcription factor A (TFAM) to elevate the content of mitochondrial DNA (mtDNA), but also facilitates the expression of autophagy-associated genes (e.g., BNIP3 and LC3), thus synergistically enhancing mitophagic activity [110,111,112]. Experimental evidence shows that AMPK inhibitors significantly block exercise-induced activation of mitophagy and up-regulation of PGC-1α in bone cells; at the same time, they inhibit the expression of osteogenic differentiation markers, and this phenomenon directly confirms that this pathway plays a central regulatory role in exercise-induced modulation of bone metabolism [113].

Mechanical stress generated during exercise is transduced into biochemical signals via mechanosensors on the cell membrane, thereby indirectly regulating mitophagy. This effect predominantly targets the osteogenic lineage and exhibits clear cell specificity [114]. Mechanosensitive molecules such as Piezo1/2 channels and polycystins (PC) located on the membranes of osteocytes and BMSCs play key roles in sensing mechanical stimuli [72]. Mechanical stress can directly activate Piezo1 channels, inducing calcium influx and subsequently activating AMPK through the Ca^2+^/CaMKII/AMPK pathway, thereby initiating mitophagy [115]. In addition, mechanical stress can upregulate the expression of sirtuin 1 (SIRT1), enhance AMPK phosphorylation, and further promote mitophagy associated with osteogenic differentiation of BMSCs [116]. Conversely, Piezo1 knockout mice exhibit impaired mechanosensation, leading to reduced mitophagy activity and mitochondrial dysfunction in bone cells, ultimately resulting in bone loss that cannot be reversed by exercise intervention. This confirms that the Piezo1/AMPK pathway is essential for exercise-induced regulation of mitophagy in bone cells [114].

Furthermore, mechanical stress can indirectly reduce oxidative stress of the bone microenvironment by upregulating anti-inflammatory factors (e.g., IL-10) [117], thereby creating favorable conditions for efficient activation of mitophagy.

In conclusion, metabolic stress and mechanical stress are deeply interconnected in the exercise-mediated regulation of mitophagy. Acting via signaling factors such as AMPK and PGC-1α, which may serve as potential targets for preventing bone loss, they jointly modulate bone remodeling.

#### 4.1.2. Exercise Regulates Adaptive Mitophagy via Hypoxia-Induced HIF-1/BNIP3 Signaling

Exercise-induced hypoxic stress can activate the HIF-1/BNIP3 signaling pathway, thereby regulating mitophagy within the bone microenvironment. High-intensity exercise or training under hypoxic conditions can induce local hypoxia in the bone microenvironment, thereby activating HIF-1. HIF-1 upregulates the expression of BNIP3, which in turn activates BNIP3-mediated mitophagy to eliminate mitochondria damaged by hypoxic stress [97,118]. In addition, HIF-1 can promote mitochondrial biogenesis by activating the MAPK–PGC-1α signaling pathway [119], forming a coordinated “mitophagy–biogenesis” regulatory axis. This synergistic mechanism enhances the adaptability of bone cells to hypoxic environments, such as those encountered at high altitude [120]. It is critical to note that hypoxia also promotes osteoclast differentiation.

Therefore, precise modulation of exercise intensity and hypoxic exposure level is critical for maximizing the positive effects of exercise interventions on bone metabolism.

#### 4.1.3. Exercise Optimizes Vitamin D/Calcium Networks and Estrogen Signaling to Enhance the Regulatory Efficiency of Mitophagy in the Bone Microenvironment

Vitamin D and calcium are core nutrients in bone metabolism and influence mitophagy through a coordinated regulatory network characterized by “vitamin D-mediated absorption and calcium homeostasis.” Exercise enhances the regulatory efficiency of this network on mitophagy by upregulating VDR expression and modulating the fibroblast growth factor 23–parathyroid hormone (FGF23–PTH) axis, thereby supporting bone remodeling homeostasis. On the one hand, exercise significantly upregulates VDR expression in bone tissue and the liver [121,122,123]. VDR is the key receptor mediating the biological effects of 1,25(OH)_2_D_3_ [32,34]. Increased VDR expression enhances its binding capacity to 1,25(OH)_2_D_3_, improves the utilization efficiency of the active form of vitamin D, and prevents excessive accumulation of 1,25(OH)_2_D_3_ that would otherwise activate CYP24A1 (an enzyme responsible for degrading 1,25(OH)_2_D_3_ and reducing its activity) [34,124]. This process ultimately strengthens vitamin D-mediated activation of mitophagy. Wang et al. [121] demonstrated that long-term aerobic exercise increased VDR expression in the bone tissue of aged rats by more than 30%, accompanied by a significant enhancement of mitophagy activity in osteoblasts. On the other hand, exercise dynamically regulates the FGF23–PTH axis to maintain calcium homeostasis and vitamin D activation [125]. During short-term exercise, increased calcium consumption transiently activates PTH and 1,25(OH)_2_D_3_, promoting calcium absorption and bone calcium mobilization to maintain serum calcium balance [126]. In contrast, long-term exercise optimizes the regulatory efficiency of this axis: in healthy individuals, it slightly increases serum calcium levels and enhances bone mineralization [127]; in osteoporotic patients, it significantly reduces pathologically elevated PTH levels, promotes calcium absorption by improving vitamin D utilization, restores calcium homeostasis, and reduces compensatory calcium release driven by excessive bone resorption [128,129]. Additionally, exercise upregulates α-Klotho (a co-receptor of FGF23), enhancing the regulatory efficiency of FGF23 on calcium and phosphate metabolism while reducing its inhibitory effect on CYP27B1 (the enzyme responsible for vitamin D activation). This helps maintain stable conversion of 1,25(OH)_2_D_3_ [34,130,131].

Collectively, by optimizing the vitamin D/calcium regulatory network, exercise indirectly enhances the function of mitophagy in preserving the balance of bone remodeling.

Estrogen is a key hormone regulating bone metabolism in females and modulates mitophagy through binding to estrogen receptors (ERs). Exercise regulates estrogen signaling through multiple pathways. First, exercise-induced upregulation of estrogen receptor expression represents a central mechanism. Animal studies have shown that progressive treadmill training (12 weeks: 10–18 m/min, 15–60 min/day, 5 days/week; and 10 weeks: 12–24 m/min, 20–60 min/day, 5 days/week) significantly increases ERα and ERβ expression in ovariectomized (OVX) rats [132,133]. Recent human studies further demonstrate that 12 weeks of moderate-intensity aerobic training (50–60 min/day, 3 days/week, 65–70% heart rate reserve) significantly upregulates ERα expression in vitamin D-deficient or obese postmenopausal women [134,135]. Second, exercise can regulate estrogen levels via the hypothalamic–pituitary–gonadal (HPG) axis. For example, four weeks of swimming training significantly reduced the levels of gonadotropin-releasing hormone (GnRH), luteinizing hormone (LH), and follicle-stimulating hormone (FSH) in Alzheimer’s disease (AD) model rats, thereby improving HPG axis dysfunction and cognitive impairment [136]. These hormonal changes are also associated with exercise-induced irisin. In vitro studies have demonstrated that irisin can elevate the expression of LH and FSH in pituitary cells [137]. It has therefore been hypothesized that irisin may exert negative feedback on GnRH or compete with GnRH for receptor binding sites, ultimately modulating estrogen levels [138]. In addition, aromatase (ARO) has recently been recognized as an important target in exercise-mediated regulation of estrogen. Eight weeks of moderate-intensity treadmill training (10–18 m/min, 60 min/day, 5 days/week) increased myogenic estradiol (E_2_) levels by 66% in OVX mice, while significantly upregulating ARO expression and activity, partially reversing mitochondrial dysfunction in skeletal muscle induced by OVX [139]. It is suggested that ARO may represent another key target for exercise-mediated prevention and intervention of osteoporosis, which warrants further experimental verification.

Overall, the impacts of exercise on estrogen are not confined to mere increases or decreases in hormone concentrations. Rather, its key role lies in improving the efficiency of estrogen signaling, thereby optimizing hormonal regulation and further enhancing the regulatory effects of mitophagy on bone remodeling.

#### 4.1.4. Differential Effects of Exercise Modalities on Mitophagy Regulation

The regulatory effects of exercise on mitophagy are not uniform or continuous. Acute exercise predominantly induces reparative mitophagy, whereas long-term exercise promotes adaptive enhancement of mitophagy. Together, these processes synergistically contribute to the sustained optimization of mitochondrial function in bone cells.

Following acute exercise (e.g., a single bout of high-intensity interval exercise or endurance exercise), mitochondria in bone cells and skeletal muscle cells undergo transient functional impairment due to sustained oxidative phosphorylation, which is accompanied by a marked increase in the production of ROS. Under these conditions, mitophagy is rapidly activated, primarily via the BNIP3-mediated ubiquitin-independent pathway [140,141]. Studies have shown that during the recovery phase (3–12 h) after acute exercise, the expression of BNIP3 and LC3-II in bone tissue is remarkably increased, whereas key molecules in the PINK1/Parkin pathway show no significant changes [142,143]. This suggests that mitophagy during this stage mainly functions to eliminate damaged mitochondria and reduce ROS accumulation, thereby creating favorable conditions for subsequent mitochondrial biogenesis [143,144]. Meanwhile, an increase in PGC-1α expression is observed 2–3 h post-exercise, further confirming the coordinated interaction between mitophagy and mitochondrial biogenesis during the repair phase [145,146,147].

In contrast, long-term exercise (e.g., 12 weeks of treadmill training or 10 weeks of swimming training) induces adaptive mitochondrial remodeling, resulting in sustained enhancement of mitophagy, with a shift toward the PINK1/Parkin-mediated ubiquitin-dependent pathway [148,149]. Long-term exercise upregulates the SIRT1/FOXO1/3 signaling pathway, thereby facilitating the expression of PINK1 and Parkin, and enhancing the efficiency of recognizing and degrading damaged mitochondria. Concurrently, long-term exercise reduces the expression of HIF1 and BNIP3 in the bone microenvironment, suggesting that mitochondrial function in bone cells has reached a stable state through adaptive remodeling and no longer relies on stress-induced BNIP3 signaling [150]. The core significance of this adaptive shift lies in the elevation of basal mitophagy activity, which enhances antioxidant capacity and stabilizes energy supply in bone cells. This provides sustained support for osteogenic differentiation and ultimately contributes to continuous improvements in bone mineral density.

In summary, mitophagy induced by acute exercise primarily supports post-exercise repair and regeneration processes (e.g., muscle fiber remodeling and mitochondrial biogenesis) (Table 1). In contrast, the promotion of bone remodeling via mitophagy requires adaptive mitochondrial responses to exercise-induced stress, including enhanced ATP production, coordinated regulation of autophagy and biogenesis, and improved resistance to oxidative stress. The chronic effects of long-term exercise fulfill the quantitative requirements for such adaptive changes (Table 1), highlighting the critical importance of sustained or lifelong exercise in promoting bone remodeling.

## 5. Conclusions and Perspectives

Exercise activates mitophagy through both direct stress-mediated signaling and indirect optimization of the bone microenvironment. Emerging evidence has begun to elucidate the central role of mitophagy in mediating exercise-induced regulation of bone remodeling. However, several critical scientific questions remain to be addressed. First, most relevant conclusions originate from cell experiments and animal models; preclinical findings are difficult to replicate in humans, and interspecies differences in bone metabolism and mitochondrial quality control limit translational application. On the one hand, the existence of a dose–response relationship between exercise and mitophagy regulation remains unclear. For example, different intensities of weight-bearing exercise (e.g., jogging vs. high-intensity interval running) may differentially influence the degree of hypoxia in the bone microenvironment. How to precisely match the activation threshold of the HIF1/BNIP3 pathway to avoid excessive osteoclast differentiation remains an open question. In addition, during long-term exercise, whether the elevation of basal mitophagy activity reaches a “ceiling effect” is still unknown, and exceeding this threshold may potentially trigger excessive mitophagy and induce apoptosis in bone cells. On the other hand, there are significant gaps in mechanistic studies involving special populations and pathological conditions. Most existing studies are based on healthy animal models or normal cell systems, whereas limited attention has been given to high-risk populations for osteoporosis (e.g., the elderly and patients with diabetes). For instance, in elderly individuals, whether reduced VDR expression attenuates the exercise-induced activation of the vitamin D/calcium–mitophagy pathway remains unclear. Similarly, under hyperglycemic conditions associated with diabetes, whether exercise-induced AMPK activation is suppressed by insulin resistance—thereby reducing the efficiency of mitophagy regulation—requires further investigation. Addressing these questions is essential for optimizing exercise-based interventions in these populations. Given the prevailing limitations of existing evidence, subsequent research should prioritize characterizing mitophagy profiles in distinct populations. Multi-omics approaches including transcriptomics and metabolomics can help identify the activation and saturation thresholds of key regulatory pathways. Dose-finding studies with molecular endpoints are essential to optimize exercise protocols, and genetic markers may support the development of personalized exercise regimens combined with wearable monitoring of exercise adherence. Targeted pharmacological or gene therapy against mitophagy pathways merits further preclinical investigation, and long-term prospective studies lasting more than 5 years are required to assess fracture-related clinical outcomes. Additionally, the mitophagy-dependent skeletal benefits induced by exercise should be explored in wider scenarios, including fracture healing, microgravity-induced bone loss, and postsurgical bone repair following bone tumor resection.

In summary, mitophagy serves as a central mediator of exercise-regulated bone remodeling. Advances in this area not only deepen insights into the exercise–bone health axis but also provide innovative therapeutic targets for the precise management of bone metabolic disorders such as osteoporosis.

## Figures and Tables

**Table 1:** Summary of studies on acute and chronic exercise-mediated mitophagy. This table summarizes representative preclinical animal experiments and human trials exploring the regulatory effects of acute and chronic exercise on mitophagy. Differences in exercise modes, experimental subjects, intervention duration, and key molecular findings across included studies are listed to illustrate the divergent mitophagic signaling responses triggered by different exercise protocols.

Author	Article	Type of Exercise	Objects	Duration	Conclusion
Li H, Miao W, Ma J, Xv Z, Bo H, Li J, et al. [141]	Acute Exercise-Induced Mitochondrial Stress Triggers an Inflammatory Response in the Myocardium via NLRP3 Inflammasome Activation with Mitophagy	Acute treadmill running	Male Sprague-Dawley rats (age 8 weeks)	/	Beclin1, LC3, and Bnip3 were all significantly upregulated during acute exercise
Drake JC, Laker RC, Wilson RJ, Zhang M, Yan Z. [143]	Exercise-induced mitophagy in skeletal muscle occurs in the absence of stabilization of Pink1 on mitochondria	Acute treadmill running	Male C57BL/6J mice (10–12 weeks old)	/	Pink1 is not involved in exercise-induced mitophagy in skeletal muscle.
Cobley JN, Bartlett JD, Kayani A, Murray SW, Louhelainen J, Donovan T, et al. [145]	PGC-1alpha transcriptional response and mitochondrial adaptation to acute exercise is maintained in skeletal muscle of sedentary elderly males.	20 min high-intensity interval (HIT) on a bicycle ergometer	12 young (23.15 ± 3.5 years old) and 12 older (64.15 ± 4.25 years old) healthy males	/	Acute exercise induced increases in PGC-1α expression at 3 h post-exercise
Zhao N, Zhang X, Li B, Wang J, Zhang C, Xu B. [148]	Treadmill Exercise Improves PINK1/Parkin-Mediated Mitophagy Activity Against Alzheimer’s Disease Pathologies by Upregulated SIRT1-FOXO1/3 Axis in APP/PS1 Mice	Treadmill exercise	Male APPswe/PS1dE9 (APP/PS1) double-transgenic mice and non-transgenic C57BL/6 J wild-type mice	45 min/day, 5 days/week, for 12 weeks	12-week treadmill exercise program improved mitochondrial function by enhancing PINK1/Parkin-mediated mitophagy activity
Zhao Y, Zhu Q, Song W, Gao B. [149]	Exercise training and dietary restriction affect PINK1/Parkin and Bnip3/Nix-mediated cardiac mitophagy in mice	Swimming training	Male C57BL/6 mice (eight weeks)	20 min/day, 5 days/week, for 10 weeks	After 10-week swimming training, both PINK1 mRNA and protein levels increased significantly, while Bnip3 and Nix expressions decreased significantly

## Data Availability

The original contributions presented in this study are included in the article. Further inquiries can be directed to the corresponding authors.

## Abbreviations

OP	Osteoporosis
ROS	Reactive Oxygen Species
BMSCs	Bone Marrow Mesenchymal Stem Cells
PINK1	PTEN-induced kinase 1
BNIP3	Bcl-2/adenovirus E1B 19 kDa-interacting protein 3
NIX	NIP3-like protein X
FUNDC1	FUN14 domain-containing protein 1
VDR	Vitamin D Receptor
1,25(OH)_2_D_3_	1,25-dihydroxyvitamin D3
SOD	Superoxide Dismutase
GSH	Glutathione
CaMKKβ	Calcium/Calmodulin-dependent Protein Kinase Kinase β
AMPK	AMP-activated Protein Kinase
mTOR	Mammalian Target of Rapamycin
ULK1	Autophagy-related Kinase 1
TFEB	Transcription Factor EB
MAMs	Mitochondria-associated Endoplasmic Reticulum Membranes
ER	Estrogen Receptor
ERα/ERβ	Estrogen Receptor α/β
GPER	G Protein-coupled Estrogen Receptor 1
SIRT1	Sirtuin 1
SIRT3	Sirtuin 3
ERK1/2	Extracellular Regulated Protein Kinases 1/2
PI3K/Akt	Phosphatidylinositol 3-Kinase/Protein Kinase B
MAPK	Mitogen-activated Protein Kinase
RANKL	Receptor Activator of Nuclear Factor-κB Ligand
ERRs	Estrogen-related Receptors
CTSK	Cathepsin K
Runx2	Runt-related Transcription Factor 2
Osx	Osterix
PPARγ	Peroxisome Proliferator-activated Receptor γ
C/EBPα	CCAAT/Enhancer Binding Protein α
HIF-1α	Hypoxia-inducible Factor-1α
NFATc1	Nuclear Factor of Activated T Cells c1
NF-κB	Nuclear Factor-κB
TRAP	Tartrate-resistant Acid Phosphatase
PGC-1α	Peroxisome Proliferator-activated Receptor Gamma Coactivator-1α
TFAM	Mitochondrial Transcription Factor A
Piezo1/2	Piezo-type mechanosensitive ion channel component 1/2
PC	Polycystins
FGF23	Fibroblast Growth Factor 23
PTH	Parathyroid Hormone
CYP24A1	Cytochrome P450 family 24 subfamily A member 1
CYP27B1	Cytochrome P450 family 27 subfamily B member 1
HPG	Hypothalamic–Pituitary–Gonadal
GnRH	Gonadotropin-releasing Hormone
LH	Luteinizing Hormone
FSH	Follicle-stimulating Hormone
ARO	Aromatase
E_2_	Estradiol
OVX	Ovariectomized
mtDNA	Mitochondrial DNA
VDAC1	Voltage-dependent Anion Channel 1
FOXO1/3	Forkhead Box O1/3
